# European *Culex pipiens* Populations Carry Different Strains of *Wolbachia pipientis*

**DOI:** 10.3390/insects15090639

**Published:** 2024-08-26

**Authors:** Tobias Lilja, Anders Lindström, Luis M. Hernández-Triana, Marco Di Luca, Olivia Wesula Lwande

**Affiliations:** 1 Department of Microbiology, Swedish Veterinary Agency, 751 89 Uppsala, Sweden; anders.lindstrom@sva.se; 2 Vector-Borne Diseases Research Group, Virology Department, Animal and Plant Health Agency (APHA), Addlestone KT15 3NB, UK; luis.hernandez-triana@apha.gov.uk; 3 Department of Infectious Diseases, Istituto Superiore di Sanità, 00161 Rome, Italy; marco.diluca@iss.it; 4 Department of Clinical Microbiology, Umeå University, 901 85 Umeå, Sweden; olivia.lwande@umu.se

**Keywords:** mosquito, *Culex pipiens*, *Wolbachia*, taxonomy, phylogenetics

## Abstract

**Simple Summary:**

The mosquito species *Culex pipiens*, has two ecotypes, pipiens and molestus. The two forms differ in mating behavior and how they manage overwintering, with the molestus form thriving in closed indoor surroundings but without an ability to manage cold winter conditions, and the pipiens form that requiring open spaces for mating and going into diapause during winter. Both ecotypes are important vectors for mosquito-borne viruses. *Cx. pipiens* mosquitoes often carry endosymbiotic *Wolbachia* bacteria that are transmitted from the mother to the egg, but this varies frequency of occurrence. The *Wolbachia* endosymbiont affects the reproductive success of mosquitoes so that only eggs infected with a compatible *Wolbachia* can be fertilized by an infected male. In artificially infected *Aedes* mosquitoes, *Wolbachia* affects whether the mosquito can transmit a virus though bites. We studied how *Cx. pipiens* mosquitoes from Sweden carried *Wolbachia* and compared them to mosquitoes from other European countries, and found that all tested *Cx. pipiens* mosquitoes carried *Wolbachia*. We characterized the *Wolbachia* from these mosquitoes and found that *Cx. pipiens* of the two ecotypes carry different strains of *Wolbachia* in Sweden and Norway but not in sampled mosquitoes from Italy or England. This highlights the differences between the two ecotypes in northern Europe.

**Abstract:**

The mosquito *Culex pipiens* occurs in two ecotypes differing in their mating and overwintering behavior: pipiens mate in open environments and diapause, and molestus also mate in small spaces and is active throughout the year. *Cx. pipiens* carry *Wolbachia* endosymbionts of the *w*Pip strain, but the frequency of infection differs between studied populations. *Wolbachia* infection affects the host reproductive success through cytoplasmic incompatibility. *w*Pip *Wolbachia* is divided into five types, *w*Pip I–V. The type of *w*Pip carried varies among *Cx. pipiens* populations. In northern European locations different *w*Pip types are found in the two ecotypes, whereas in southern locations, they often carry the same type, indicating differences in hybridization between ecotypes. In this study, *Cx. pipiens* specimens of both ecotypes were collected from Sweden and compared to specimens from Norway, England, Italy, and the Netherlands, as well as *Cx. quinquefasciatus* from Mali and Thailand. The abundance varied, but all specimens were infected by *Wolbachia*, while the tested specimens of other mosquito species were often uninfected. The *w*Pip strains were determined through the sequence analysis of *Wolbachia* genes *ank2* and *pk1*, showing that *Cx. pipiens* ecotypes in Scandinavia carry different *w*Pip strains. The observed differences in *w*Pip strains indicate that hybridization is not frequent and may contribute to barriers against hybridization of the ecotypes in Sweden and Norway.

## 1. Introduction

The mosquito species *Culex pipiens* is established in large parts of the world and is part of a species complex with *Culex quinquefasciatus* [1]. Within the species *Culex pipiens,* there are two ecotypes, *Cx. pipiens* form (f) pipiens and *Cx. pipiens* f molestus, that differ in terms of their behavior, whereby the pipiens form mates above ground, is non-autogenous, diapauses in winter, and is ornithophilic, while the molestus form can mate underground, is autogenous, does not diapause, and readily also bites mammals, as reviewed in [2]. The two ecotypes are defined by their behavioral characteristics and cannot be reliably differentiated morphologically [3]. Microsatellites throughout the genome have been used to separate the populations. One marker, the CQ11 microsatellite, is fixed as one allele in the molestus form and a separate allele is fixed in the pipiens form, with hybrids being heterozygous, which makes it useful to identify the ecotypes [4]. While the two ecotypes can be clearly separated in northern European countries, there is more hybridization observed between the two forms in the Mediterranean region [2].

The *Cx. pipiens* complex is an important vector of pathogens, including viruses such as the West Nile virus and Usutu virus, and protozoans such as avian malaria [5,6,7]. The complex has also been shown to carry strains of *Wolbachia*, an intracellular endosymbiotic bacterium that is transovarially transmitted from females to offspring. *Wolbachia* can affect the reproductive success of the infected mosquitoes by cytoplasmic incompatibility (CI), a mechanism that affects the sperm in such a way that only the eggs infected with a compatible strain of *Wolbachia* can be fertilized and develop normally [8], while uninfected eggs or eggs infected by a non-compatible strain of *Wolbachia* that are fertilized by sperm from an infected male will fail to develop. Despite *Wolbachia* affecting the gametes of both sexes, it is only transferred to the egg from the female [9]. In *Aedes aegypti*, *Wolbachia* infection has been seen to affect the vector competence, and an infection of *Ae. aegypti* with specific *Wolbachia* strains has been developed into new strategies to combat dengue virus infections [10]. On the other hand, natural *Wolbachia* infections affecting vectorial capacity has been an active area of research.

In all tested populations of *Cx. pipiens*, infection with *Wolbachia* of the *w*Pip strain is highly prevalent, but the proportion of mosquitoes infected differs between studies. Some studies found that all individual mosquitoes carry *Wolbachia* [11,12]. Californian *Cx. pipiens* showed close to 100% infection rate. The study also reported vertical transmission rates in laboratory strains and wild populations and found that over 98% of embryos were infected [13]. Whether uninfected embryos were viable or not was not estimated. Other studies showed lower infection rates. In a previous Swedish study, 97% of *Cx. pipiens* tested were infected by *w*Pip *Wolbachia* [14]. In a Russian study, underground populations of *Cx. pipiens*, which are most likely *Cx. pipiens* f molestus, had infection rates of as low as 70%, while outdoor populations had infection rates close to 100% [15]. A Belarusian study also found uninfected individuals of both ecotypes, where some populations had infection rates under 50% [16]. In German samples, about 93% of all tested *Cx. pipiens* f pipiens were positive for *Wolbachia* [17]. In a study from Iran, *Wolbachia* DNA was found in 87% of 260 wild-caught mosquitoes. The rate of infection in adult females ranged from 61% to 100%, and in males from 80% to 100% [18]. Also, a Chinese study showed that infection rates differed between locations, from no *Wolbachia*-infected individuals to 100% infected [19]. This study even confirmed negative samples with a secondary PCR method to rule out false negatives [19], showing that *Cx. pipiens* populations can be uninfected.

The *Wolbachia* present in *C. pipiens* are all from the *w*Pip group. Studies of many diverse *w*Pip *Wolbachia* show that this group is monophyletic [20], but within the *w*Pip strain of *Wolbachia,* there is considerable genetic diversity. The *w*Pip *Wolbachia* group can be divided into five distinct groups *w*Pip I-V. Using *Cx. pipiens* specimens from many parts of the world, the classification of *w*Pip groups was described from the sequence variation in a concatenated sequence from seven genes in the *w*Pip genome, *MutL, ank2*, *pk1*, *pk2*, *GP12*, *GP15*, and *RepA*. [20]. While there are many recombinations between strains, these genes mostly follow the same pattern, and the *w*Pip type has later been implicated using restriction fragment length polymorphism (RFLP) of *ank2* and *pk1* [21]. The ank2 and pk1 markers are in genes containing ankoryn motifs that are thought to mediate protein–protein interactions [22]. The *w*Pip groups have also been observed to correlate with the mitochondrial genotype [20], indicating that the *Wolbachia* strain and mitochondria are both predominantly inherited maternally with no major contributions from horizontal transmission. The nuclear genome on the other hand did not show the same pattern and similar *w*Pip groups and mitochondria could be carried both by *Cx. pipiens* and *Cx. quinquefasciatus* [20]. Similarly, the same *w*Pip group has been found in both pipiens and molestus ecotypes [23]. A major reason for determining the *w*Pip group in specimens is to try to infer if there is cytoplasmic incompatibility between specimens. *Cx. pipiens* specimens carrying *Wolbachia* from the same *w*Pip group are more likely to be compatible while specimens carrying different *w*Pip groups are more likely to have at least partial incompatibility. However, determining the *w*Pip group is not enough to fully determine the CI pattern [11,21].

The type of *w*Pip *Wolbachia* carried has been studied in several *Cx. pipiens* populations, revealing a wide variation within and between locations. For example, in northern locations such as Moscow and Volgograd, Russia, as well as in Berlin and Hannover, Germany, *w*Pip II was found in the pipiens ecotype while *w*Pip IV was found in the molestus ecotype. *w*Pip I and *w*Pip II were found in both ecotypes in Comport, Portugal, and in Prades le Lez, France, respectively [23]. In Morocco, *w*Pip I, IV, and V are all present and both *w*Pip I and V were found in both ecotypes [24]. In Turkey, *w*Pip I and II dominated but IV was found in two locations in Northwestern Turkey. The study did not differentiate between ecotypes, but all three *w*Pip groups were found in both rural and urban sites [11]. From the published results, it seems that *w*Pip groups (and mitochondrial genomes) are different in the two ecotypes in northern locations, whereas there is more hybridization in more southern locations [11,23,24]. 

In addition to the CI effects the different *w*Pip strains may have, they can also affect the biology of the mosquito in different ways. *Cx. quinquefasciatus* carrying their natural *w*PipSJ *Wolbachia* is less susceptible to the pathogenic action of mosquitocidal bacterial strains when compared with antibiotic-treated mosquitoes that do not carry *Wolbachia* [25]. The response to viral infections may also be affected by *w*Pip strains. The infection load of *Culex pipiens* densovirus (CxDV), which is an insect-specific virus that is vertically transmitted in *Cx. pipiens*, correlated with the *Wolbachia* load. There was further difference depending on the *w*Pip type, where *w*Pip I was associated with higher viral loads than *w*Pip IV [26]. In Tunisia, where both *w*Pip I and *w*Pip IV are present, areas with one *w*Pip type also had specific CxDV strains, indicating that they are inherited together [27]. Whether differences between *w*Pip groups also can affect coinfection with other viruses has not been tested.

Since the infection rate in *Cx. pipiens* has differed between previous reports and some reports have suggested that *Wolbachia* infection rates differ between the two ecotypes, we wanted to investigate what proportion of the different mosquito populations carried *Wolbachia* and whether that proportion differed between the ecotypes. We analyzed the infection level of *Wolbachia* in *Cx. pipiens* mosquitoes from Sweden and Norway and compared them with mosquitoes from other locations, as well as other mosquito species. To better characterize the tested populations and see potential differences, we investigated which *w*Pip strains infect *Cx. pipiens* of both ecotypes collected in different regions through sequence analysis of the *Wolbachia* genes *ank2* and *pk1*.

## 2. Materials and Methods

**Figure 1:**
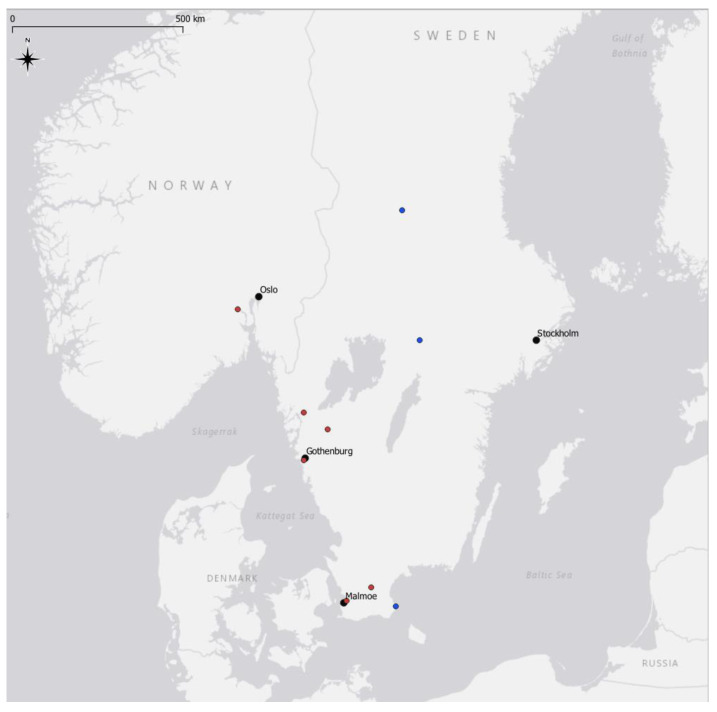
Collection sites for the *Cx. pipiens* specimens collected in Sweden and Norway. Blue dots are collection sites for *Cx. pipiens* f pipiens specimens and red dots are collection sites for *Cx. pipiens* f molestus specimens.

*Mosquito collection: Cx. pipiens* f molestus mosquitoes from Sweden and Norway were collected through citizen science where people experiencing nuisance collected mosquitoes. The Swedish samples were collected in Gothenburg, Sollebrunn, Uddevalla, Malmoe and Hoorby. The Norwegian samples were collected in Drammen. *Cx. pipiens* f pipiens mosquitoes from Sweden were collected with BG sentinel traps in Gothenburg (Transsafe project) and overwintering locations (Örebro) with mosquito magnet traps (Mosquito Magnet, Lancaster, PA, USA) (Simrishamn) (Figure 1). *Aedes vexans*, *Culex modestus*, *Culex territans,* and *Culex torrentium* mosquitoes were collected in Sweden as part of a citizen science project (Fånga myggan, https://www.sva.se/aktuellt/pressmeddelanden/fanga-myggan/, accessed on 1 August 2024) urging citizens to send mosquitoes from different parts of Sweden. Mosquitoes and extracted DNAs were also provided by collaborators for mosquitoes collected in Italy and England, and lab strains originating from England, the Netherlands, and Thailand. *Culex quinquefasciatus* from Mali were collected with BG sentinel traps (Biogents, Regensburg, Germany). In total, 192 mosquitoes, including 154 *Cx. pipiens*, 8 *Cx. quinquefasciatus*, 9 *Culex torrentium*, 6 *Culex modestus*, 2 *Culex territans,* and 13 *Aedes vexans,* were tested for *Wolbachia* presence by qPCR wsp amplification. 

DNA extraction from mosquitoes: To assess whether *Wolbachia* was spread throughout the body, the mosquitos were separated into body parts including, the abdomen and thorax with the head, wings, and legs, respectively. Tissues were homogenized using a plastic pestle and DNA was extracted separately from each part using the QIAamp DNA Mini Kit (QIAGEN, Hilden, Germany) following the manufacturer’s instructions. DNA was eluted in 200 µL.

Real-time PCR identification of Cx. pipiens ecotypes: Differentiation of *Cx. torrentium* and *Cx. Pipiens,* and further differentiation of *Cx. pipiens* specimens to ecotype were performed as previously described [28]. Shortly, to distinguish *Cx. torrentium*, species-specific primers and probe amplifying part of the *Ace2* locus were used. To separate *Cx. pipiens* f pipiens and *Cx. pipiens* f molestus, primers and probes recognizing the different CQ11 alleles were used. All qPCRs were set up using Perfecta Toughmix 2x (QuantaBio, Beverly, MA, USA) and 2 µL mosquito DNA extract.

Real-time PCR detection of Wolbachia: The level of *Wolbachia* infection in the abdomen and thorax was determined by qPCR, as previously described [17]. The primers used were Wol_wsp_OSM_323(5′-TAGCGATTGAAGATATGC) and Wol_wsp_OSM_324(5′-CTAGCTTCTGAAGGATTG), each at 0.6 µM, the probe Wol_wsp_probe_OSM_324(5’/56-FAM/CACCAACAC/ZEN/CAACACCAA) was used at 0.02 µM [17]. The PCR was set up using Perfecta Toughmix 2× (QuantaBio, Beverly, MA, USA) and 2 µL mosquito DNA extract was used. All samples were run as either duplicates or triplicates. The program was as follows: 95 °C 3 min, 95 °C 5 s, 55 °C 20 s, 72 °C 30 s, 45×. *Wolbachia* levels were normalized using a dilution series of a *Cx. pipiens* sample with high levels of *Wolbachia* included in all qPCR runs. This ensured that the reaction was linear and not inhibited. The undiluted sample was set to a value of 1000, and this was used to create a relative quantification of *Wolbachia* in all samples.

Student’s *t*-test was used to compare levels of detected *Wolbachia* in the different populations; *p* < 0.05 was used.

PCR for sequencing: Only abdomen samples with a clearly positive *Wolbachia* detection were used for sequencing. 

Of the *pk1* gene, 1328 bp was amplified using the primers Wol_pk1_Wpa_0256_f(5′-CCACTACATTGCGCTATAGA) and Wol_pk1_Wpa_0256_r(5′-ACAGTAGAACTACACTCCTCCA) [22] at 0.4 µM. PCR was performed with AmpliTaq GoldTM DNA Polymerase with Buffer I (Thermo Fisher scientific, Waltham, MA, USA) and 1 µL of DNA. The PCR program was 95 °C 10 min, 95 °C 15 s, 52 °C 30 s, 72 °C 1.5 min ×35, 72 °C 5 min, 4 °C. Around 300 to 500 bp (deletions in some strains affect the length) of the *ank2* gene was amplified with the primers: Wol_ank2_Wpa_0652_f CTTCTTCTGTGAGTGTACGT, Wol_ank2_Wpa_ 0652_r: TCCATATCGATCTACTGCGT [22]. The products were inspected on agarose gel and sequenced using the same primers at Macrogen (Amsterdam, The Netherlands). Usable sequences were generated from 107 specimens for *ank2* and 79 specimens for *pk1*. Sequences are deposited in genbank PP690552-PP690630 (*pk1*) and PP797897-798003 (*ank2*).

Tree construction: All usable sequences were aligned using Clustal W implemented in MEGA 11 [29]. The method for evolutionary distance was selected by using the model selector in MEGA, showing that the Hasegawa–Kishino–Yano model should be used. Phylogenetic trees were calculated using the maximum likelihood method. The tree is drawn to scale and the evolutionary distances were computed using the Hasegawa–Kishino–Yano model [30], and are in the units of the number of base substitutions per site. Bootstrapping to evaluate branch support was performed with 500 iterations. Phylogenies were calculated in MEGA11.

## 3. Results

All 154 tested *Cx. pipiens* mosquitoes, regardless of the ecotype, were positive for *Wolbachia* infection as tested by wsp qPCR, while no *Wolbachia* was detected in *Aedes vexans*, *Culex territans,* or *Culex torrentium*. *Wolbachia* was detected in four of the six tested *Culex modestus* specimens, but at a much lower abundance than seen in the *Cx. pipiens* samples (Table 1).

The abundance of *Wolbachia* in each specimen was assessed in the abdomen and the rest of the body. The abundance of *Wolbachia* in the abdomen was compared between populations, but no differences were significant (Supplemental Figure S1).

**Figure 2:**
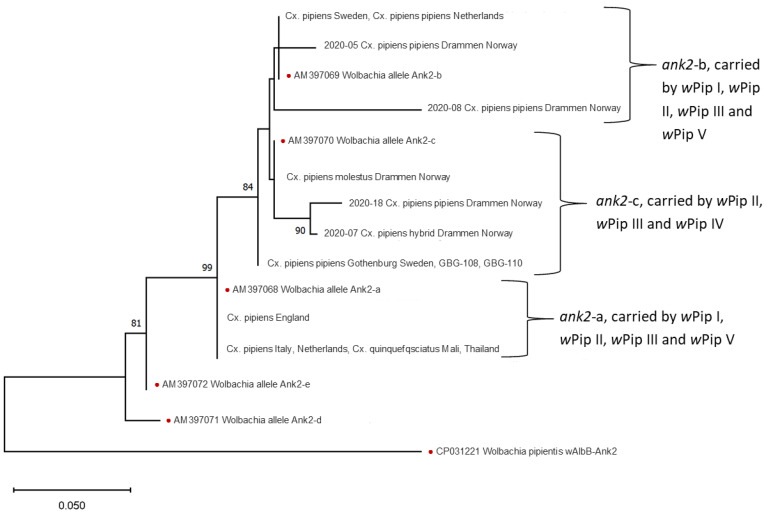
Maximum likelihood phylogenetic tree of *ank2* sequences. Specimens with identical sequences are presented together. Dots mark the previously published reference sequences [22] used to define the haplogroups and the outgroup *w*AlbB. Branches supported by bootstrap values over 70% are indicated with the bootstrap value.

To identify which strain of *Wolbachia* infected the different analyzed populations, *ank2* and *pk1* genes were amplified and sequenced from all specimens. In the *ank2* sequence, there were deletion differences between the specimens, as well as SNP differences. For *ank2* (Figure 2), all Swedish specimens, except two *Cx. pipiens* f pipiens specimens from Gothenburg, grouped together with *Cx. pipiens* f pipiens from Norway and the Netherlands, but were separate from specimens from the other locations. For the Swedish specimens, this marker was not differentiating between the two ecotypes. However, the Norwegian *Cx. pipiens* f molestus specimens did group separately from the other specimens for this marker.

Italian and English specimens of both ecotypes grouped together with the *Cx. quinquefasciatus* specimens and the *Cx. pipiens* f. molestus from the Netherlands.

**Figure 3:**
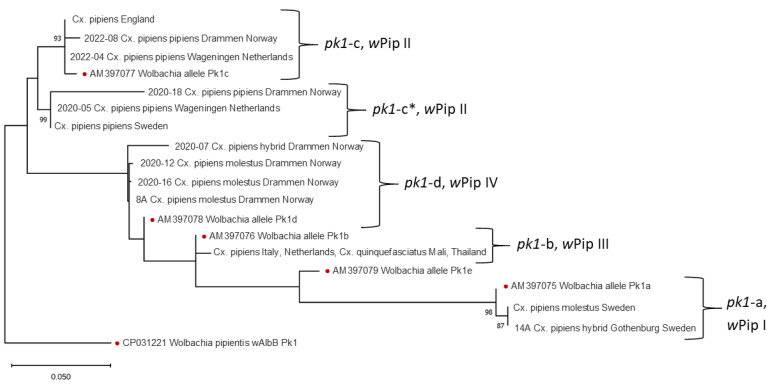
Maximum likelihood phylogenetic tree of *pk1* sequences. Specimens with identical sequences are presented together. Dots mark the previously published reference sequences [22] that are used to define the haplogroups and the outgroup *w*AlbB. The *Cx. pipiens* f pipiens specimens from Sweden and Norway group together in a distinct cluster (*pk1*-c*) not defined as a haplogroup in [22]. Branches supported by bootstrap values over 70% are indicated with the bootstrap value.

By *pk1* analysis (Figure 3), Swedish specimens grouped according to ecotype with all *Cx. pipiens* f molestus and one hybrid grouping together with the *pk1*-a allele. The *Cx. pipiens* f pipiens specimens formed a clade with one specimen from the Netherlands and one from Norway. This clade was not directly represented by any of the *pk1* alleles described previously [12] but is closest to the *pk1*-c allele. The English specimens as well as one of the Norwegian *Cx. pipiens* f pipiens and a *Cx. pipiens* f pipiens from the Netherlands all grouped with the *pk1*-c allele. The Norwegian *Cx. pipiens* f molestus specimens and a hybrid specimen grouped together with the *pk1*-d allele. The Italian specimens as well as the *Cx. quinquefasciatus* and the molestus form from the Netherlands all grouped with the *pk1*-b allele.

These results show that the pipiens and the molestus ecotypes in Scandinavia carry different strains of *w*Pip. Specimens from England and Italy share the same strain of *w*Pip, regardless of ecotype. Similarly, English lab strains and lab strains from Netherlands and Thailand share the same *w*Pip strain. The two tested hybrids from Scandinavia, one from Drammen and one from Gothenburg, both share the *w*Pip type with the molestus specimens from the same locations. The results also show that Scandinavian *Cx. pipiens* have different *w*Pip strains from specimens from England and Italy. Using the phylogenetic tree of *ank2* and *pk1*, each specimen was designated to a *w*Pip type (Supplementary Table S1) using the same method as Dumas et al. [12]. *w*Pip-I was only found in the specimens from Swedish molestus; *w*Pip-II was found in *Cx. pipiens* f pipiens from Sweden, Norway and the Netherlands but also in both ecotypes from England. *w*Pip-III was found in both ecotypes from Italy and *Cx. pipiens* f molestus from the Netherlands and *Cx. quinquefasciatus* from Mali and Thailand. In our study, *w*Pip-IV was found in Norwegian *Cx. pipiens* f molestus.

## 4. Discussion

*Wolbachia* of the *w*Pip strain was found in all *Cx. pipiens* mosquitoes in our study. In the published literature, there are large variations in prevalence of *Wolbachia* infection in *Cx. pipiens* populations, and some studies report differences between the two ecotypes in infection prevalence. Specimens found in Africa but also in Europe that lack *Wolbachia* and have a distinct mitochondrial lineage, have been proposed to be a distinct species, *Culex juppi* [31]. In this study, all *Cx. pipiens* specimens carried *Wolbachia*, as determined by wsp qPCR, but the abundance varied. The *Wolbachia* abundance can vary greatly in *Cx. pipiens* and has even been correlated to insecticide resistance [32]. How the studies showing absence of *Wolbachia* in *Cx. pipiens* could be interpreted varies between studies. For some studies using conventional PCR methods, it is possible that the documented absence of the symbiont in some specimens depended on the lower sensitivity of the method. However, studies using similar methods differ, and Yang et al. [19] even confirmed the absence with a secondary primer set, suggesting that infection with *w*Pip *Wolbachia* may not be universal in *Cx. pipiens*. It is possible that there are specific environmental factors that affect *Wolbachia* presence, but our study did not identify any areas suitable to such studies.

The typing of *w*Pip was originally carried out dependent on a concatenated sequence of six genes, *MutL*, *ank2*, *pk1*, *pk2*, *GP12*, and *GP15* [18], and while there is evidence of recombination, these genes mostly follow the same pattern and the *w*Pip type has been implicated using the RFLP of *ank2* and *pk1* [19]. In our study, we sequenced both *ank2* and *pk1* to discover local variants within the *w*Pip types. For *ank2,* the Swedish specimens of both ecotypes grouped together, except two specimens. This might represent a less frequent allele present in parts of Sweden that was only found twice in our study.

The results from *pk1* highlighted the difference between Swedish pipiens and molestus ecotypes. Compared to *ank2*, there was more diversity in the *pk1* sequence differentiating the populations further. From the *pk1* phylogenetic tree, it was possible to see further differences than the *w*Pip types available, and it is possible to further subdivide *Wolbachia w*Pip types into separate lineages. It is clear from our results that the *w*Pip groups are mostly dependent on *pk1* sequence [12]. 

The difference in *w*Pip type between Scandinavian pipiens and molestus ecotypes indicates a stronger barrier against hybridization in Scandinavia compared to other locations, in line with the evidence reviewed by Haba and McBride [2]. Furthermore, it is possible that the *w*Pip strains themselves may reinforce this barrier between populations with cytoplasmic incompatibility. Haba and McBride [2] raise this possibility, but there are no documented cases of cytoplasmic incompatibility between the ecotypes. Swedish *Cx. pipiens* f molestus are *w*Pip-I and Swedish *Cx. pipiens* f pipiens are *w*Pip-II (special clade). In other studies, *w*Pip type I and II are compatible in some cases [21]. Often, when lines of *Cx. pipiens* carrying different lines of *w*Pip *Wolbachia* are crossed, there are examples of both unidirectional incompatibility and bidirectional incompatibility, even between lines with the same *w*Pip type. Between *w*Pip types, there is more often at least unidirectional incompatibility [21]. 

Although the rate of hybrids between ecotypes was low in our collections of *Cx. pipiens* where both ecotypes occur, we still found some hybrids which shared the *w*Pip type with the *Cx. pipiens* f molestus collected in the same area, *w*Pip-IV in Drammen and *w*Pip-I in Gothenburg. Thus, the *w*Pip types carried by the *Cx. pipiens* populations are at least permissive to cross with these lines in the female. These two hybrid specimens show that hybridization can occur as a result of molestus-form females mating with pipiens-form males. Previous studies of hybridization between the ecotypes have speculated that the most common would be the opposite with molestus-form males expanding to outdoor environments and mating with pipiens-form females [33].

Our results are compatible with the general understanding that the molestus form is spread through human trade and is separate from the local pipiens form [2,34]. Given the hypothesis that the molestus form is introduced in Scandinavian settings through human trade, molestus form in Norway has a different source from the populations in Sweden which are spread in several cities and smaller communities more than 300 km apart yet still share the same *w*Pip type. 

Many studies have shown that the pipiens and molestus forms have limited geneflow between each other in northern Europe but overlap in southern Europe and Northern Africa, reviewed by Haba and McBride [2]. In a German study, there was no geneflow detected between a lab strain of *Cx. pipiens* f molestus and field-caught *Cx. pipiens* f pipiens, even from the same area from which the lab strain was founded, suggesting that also in Germany there was little hybridization between the ecotypes [35]. A study carried out in Italy characterizing 55 populations of *Cx. pipiens* using CQ11 found that underground populations were homozygous for the f molestus allele of CQ11, and other populations had the molestus allele, heterozygotes and the pipiens allele in different proportions, with few rural populations only having the pipiens allele. Autogenous colonies could have both CQ11 alleles but the frequency of the molestus form increased during establishment of the colony but without reaching homozygosity [36]. In Moroccan *Cx. Pipiens,* there was no correlation between the trait of autogeny which is associated with the molestus ecotype and the CQ11 marker which has often been used as a proxy for the ecotype, meaning that there was such a substantial gene flow between the populations that the proxy of CQ11 genotype was no longer informative [37].

Looking mostly at *Cx. pipiens* outside of urban areas, microsatellite data do not identify *Cx. pipiens* from northern Europe as different from southern populations [38], while *w*Pip strains are clearly different in different parts of Europe [12]. This pattern highlights the difference in hereditary pattern between *Wolbachia* and mitochondria being inherited on the maternal side only and nuclear microsatellite loci that are inherited from both males and females. The difference between genetic variation in the nuclear genome of the mosquito host and the symbiont is thought to be explained by a selective sweep introducing specific variants of the *Wolbachia* symbiont [31]. 

With a growing concern for diseases spread by *Cx. pipiens*, such as West Nile fever and Usutu, also in northern Europe, the differences between the urban *Cx. pipiens* f molestus ecotype and the more rural *Cx. pipiens* f pipiens are of interest. This study used *Wolbachia w*Pip group to show that different populations of *Cx. pipiens* are infected by different *Wolbachia* and likely do not intermix to any large degree. These results further show that different populations of *Cx. pipiens* f molestus most likely have different origins and are dependent on human transports. 

## Figures and Tables

**Table 1:** Mosquitoes tested for *Wolbachia* infection by wsp qPCR. All tested *Cx. pipiens* were positive while other species had a lower incidence of infection or were completely uninfected.

Species	Location	% Wolbachia Incidence	# Wolbachia Positive	# Screened
*Aedes vexans*	Sweden	0%	0	13
*Culex modestus*	Sweden	67%	4	6
*Culex territans*	Sweden	0%	0	2
*Culex torrentium*	Sweden	0%	0	9
*Culex quinquefasciatus*	Mali, Thailand	100%	8	8
*Culex pipiens* f pipiens	Sweden	100%	35	35
*Culex pipiens* f pipiens	Norway	100%	3	3
*Culex pipiens* f pipiens	England	100%	10	10
*Culex pipiens* f pipiens	Italy	100%	24	24
*Culex pipiens* f pipiens	The Netherlands	100%	3	3
*Culex pipiens* hybrids	Sweden, Norway, England	100%	8	8
*Culex pipiens* f molestus	Sweden	100%	21	21
*Culex pipiens* f molestus	Norway	100%	5	5
*Culex pipiens* f molestus	England	100%	6	6
*Culex pipiens* f molestus	Italy	100%	36	36
*Culex pipiens* f molestus	The Netherlands	100%	3	3

# Number of specimens.

## Data Availability

Generated DNA Sequences are deposited in genbank PP690552-PP690630 (*pk1*) and PP797897-798003 (*ank2*). qPCR results are summarized in supplemental material.

## Supplementary Materials

The following supporting information can be downloaded at: https://www.mdpi.com/article/10.3390/insects15090639/s1, Table S1: sample information. Figure S1: *Wolbachia* abundance in specimens.

## References

[1] Linthicum K.J. (2012). Summary of the Symposium Global Perspective on the *Culex pipiens* Complex in the 21st Century: The Interrelationship of *Culex pipiens*, *quinquefasciatus*, *molestus* and Others. J. Am. Mosq. Control Assoc..

[2] Haba Y., McBride L. (2022). Origin and status of *Culex pipiens* mosquito ecotypes. Curr. Biol..

[3] Harbach R.E. (2012). *Culex pipiens*: Species versus species complex–taxonomic history and perspective. J. Am. Mosq. Control Assoc..

[4] Bahnck C.M., Fonseca D.M. (2006). Rapid assay to identify the two genetic forms of *Culex (Culex) pipiens* L. (Diptera: Culicidae) and hybrid populations. Am. J. Trop. Med. Hyg..

[5] Martinet J.-P., Ferté H., Failloux A.-B., Schaffner F., Depaquit J. (2019). Mosquitoes of north-western Europe as potential vectors of arboviruses: A review. Viruses.

[6] Otranto D., Dantas-Torres F., Brianti E., Traversa D., Petrić D., Genchi C., Capelli G. (2013). Vector-borne helminths of dogs and humans in Europe. Parasites Vectors.

[7] Huijben S., Schaftenaar W., Wijsman A., Paaijmans K., Takken W., Takken W., Knols B.G. (2007). Avian malaria in Europe: An emerging infectious disease?. Emerging Pests and Vector-Borne Diseases in Europe: Ecology and Control of Vector-Borne Diseases.

[8] Werren J.H., Baldo L., Clark M.E. (2008). *Wolbachia*: Master manipulators of invertebrate biology. Nat. Rev. Microbiol..

[9] Duron O., Weill M. (2006). *Wolbachia* infection influences the development of *Culex pipiens* embryo in incompatible crosses. Heredity.

[10] Ogunlade S.T., Meehan M.T., Adekunle A.I., Rojas D.P., Adegboye O.A., McBryde E.S. (2021). A review: *Aedes*-borne arboviral infections, controls and *Wolbachia*-based strategies. Vaccines.

[11] Altinli M., Gunay F., Alten B., Weill M., Sicard M. (2018). *Wolbachia* diversity and cytoplasmic incompatibility patterns in *Culex pipiens* popu-lations in Turkey. Parasites Vectors.

[12] Dumas E., Atyame C.M., Milesi P., Fonseca D.M., Shaikevich E.V., Unal S., Makoundou P., Weill M., Duron O. (2013). Population structure of *Wolbachia* and cytoplasmic introgression in a complex of mosquito species. BMC Evol. Biol..

[13] Rasgon J.L., Scott T.W. (2003). *Wolbachia* and cytoplasmic incompatibility in the California *Culex pipiens* mosquito species complex: Parameter estimates and infection dynamics in natural populations. Genetics.

[14] Bergman A., Hesson J.C. (2021). *Wolbachia* prevalence in the vector species *Culex pipiens* and *Culex torrentium* in a Sindbis virus-endemic region of Sweden. Parasites Vectors.

[15] Vinogradova E.B., Shaikevich E.V., Ivanitsky A.V. (2007). A study of the distribution of the *Culex pipiens* complex (Insecta: Diptera: Culicidae) mosquitoes in the European part of Russia by molecular methods of identification. Comp. Cy-Togenet.

[16] Khrabrova N.V., Bukhanskaya E.D., Sibataev A.K., Volkova T.V. (2009). The distribution of strains of endosymbiotic bacteria *Wolbachia pipientis* in natural populations of *Culex pipiens* mosquitoes (Diptera: Culicidae). Eur. Mosq. Bull..

[17] Leggewie M., Krumkamp R., Badusche M., Heitmann A., Jansen S., Schmidt-Chanasit J., Tannich E., Becker S.C. (2018). *Culex torrentium* mosquitoes from Germany are negative for *Wolbachia*. Med. Vet. Entomol..

[18] Karami M., Moosa-Kazemi S.H., Oshaghi M.A., Vatandoost H., Sedaghat M.M., Rajabnia Hosseini M., Maleki-Ravasan N., Yahyapour Y., Ferdosi-Shahandashti E. (2016). *Wolbachia* endobacteria in natural populations of *Culex pipiens* of Iran and its phylogenetic congruence. J. Arthropod-Borne Dis..

[19] Yang Y., He Y., Zhu G., Zhang J., Gong Z., Huang S., Lu G., Peng Y., Meng Y., Hao X. (2021). Prevalence and molecular characterization of *Wolbachia* in field-collected *Aedes albopictus*, *Anopheles sinensis*, *Armigeres subalbatus*, *Culex pipiens* and *Cx*. tritaeniorhynchus in China. PLoS Neglected Trop. Dis..

[20] Atyame C.M., Delsuc F., Pasteur N., Weill M., Duron O. (2011). Diversification of *Wolbachia* endosymbiont in the *Culex pipiens* mosquito. Mol. Biol. Evol..

[21] Atyame C.M., Labbe P., Dumas E., Milesi P., Charlat S., Fort P., Weill M. (2014). *Wolbachia* divergence and the evolution of cytoplasmic incompatibility in *Culex pipiens*. PLoS ONE.

[22] Duron O., Boureux A., Echaubard P., Berthomieu A., Berticat C., Fort P., Weill M. (2007). Variability and expression of ankyrin domain genes in *Wolbachia* variants infecting the mosquito *Culex pipiens*. J. Bacteriol..

[23] Shaikevich E.V., Vinogradova E.B., Bouattour A., Gouveia de Almeida A.P. (2016). Genetic diversity of *Culex pipiens* mosquitoes in distinct populations from Europe: Contribution of *Cx. quinquefasciatus* in Mediterranean populations. Parasites Vectors.

[24] Tmimi F.Z., Bkhache M., Mounaji K., Failloux A.B., Sarih M. (2017). First report of the endobacteria *Wolbachia* in natural populations of *Culex pipiens* in Morocco. J. Vector Ecol..

[25] Díaz-Nieto L.M., Gil M.F., Lazarte J.N., Perotti M.A., Berón C.M. (2021). Culex quinquefasciatus carrying *Wolbachia* is less susceptible to entomopathogenic bacteria. Sci. Rep..

[26] Altinli M., Soms J., Ravallec M., Justy F., Bonneau M., Weill M., Gosselin-Grenet A., Sicard M. (2019). Sharing cells with *Wolbachia*: The transovarian vertical transmission of *Culex pipiens* densovirus. Environ. Microbiol..

[27] Altinli M., Lequime S., Atyame C., Justy F., Weill M., Sicard M. (2020). *Wolbachia* modulates prevalence and viral load of *Culex pipiens* densoviruses in natural populations. Mol. Ecol..

[28] Vogels C.B., van de Peppel L.J., van Vliet A.J., Westenberg M., Ibañez-Justicia A., Stroo A., Buijs J.A., Visser T.M., Koenraadt C.J. (2015). Winter activity and aboveground hybridization between the two biotypes of the West Nile virus vector *Culex pipiens*. Vector-Borne Zoonotic Dis..

[29] Tamura K., Stecher G., Kumar S. (2021). MEGA 11: Molecular Evolutionary Genetics Analysis Version 11. Mol. Biol. Evol..

[30] Hasegawa M., Kishino H., Yano T.A. (1985). Dating of the human-ape splitting by a molecular clock of mitochondrial DNA. J. Mol. Evol..

[31] Dumas E., Atyame C.M., Malcolm C.A., Le Goff G., Unal S., Makoundou P., Pasteur N., Weill M., Duron O. (2016). Molecular data reveal a cryptic species within the *Culex pipiens* mosquito complex. Insect Mol. Biol..

[32] Berticat C., Rousset F., Raymond M., Berthomieu A., Weill M. (2002). High *Wolbachia* density in insecticide–resistant mosquitoes. Proc. R. Soc. London. Ser. B Biol. Sci..

[33] Gomes B., Sousa C.A., Novo M.T., Freitas F.B., Alves R., Côrte-Real A.R., Salgueiro P., Donnelly M.J., Almeida A.P.G., Pinto J. (2009). Asymmetric introgression between sympatric molestus and pipiens forms of *Culex pipiens* (Diptera: Culicidae) in the Comporta region, Portugal. BMC Evol. Biol..

[34] Fonseca D.M., Keyghobadi N., Malcolm C.A., Mehmet C., Schaffner F., Mogi M., Fleischer R.C., Wilkerson R.C. (2004). Emerging vectors in the *Culex pipiens* complex. Science.

[35] Honnen A.C., Monaghan M.T. (2017). City-Dwellers and Country Folks: Lack of Population Differentiation Along an Urban-Rural Gradient in the Mosquito *Culex pipiens* (Diptera: Culicidae). J. Insect Sci..

[36] Di Luca M., Toma L., Boccolini D., Severini F., La Rosa G., Minelli G., Bongiorno G., Montarsi F., Arnoldi D., Capelli G. (2016). Ecological distribution and CQ11 genetic structure of *Culex pipiens* complex (Diptera: Culicidae) in Italy. PLoS ONE.

[37] Arich S., Haba Y., Assaid N., Fritz M.L., McBride C.S., Weill M., Taki H., Sarih M., Labbé P. (2022). No association between habitat, autogeny and genetics in Moroccan *Culex pipiens* populations. Parasites Vectors.

[38] Lõhmus M., Lindström A., Björklund M. (2012). How often do they meet? Genetic similarity between European populations of a potential disease vector *Culex pipiens*. Infect. Ecol. Epidemiol..

