# Supplementary material for: European Culex pipiens Populations Carry Different Strains of Wolbachia pipientis
Source: Insects. 2024 Aug 26;15(9):639. doi: 10.3390/insects15090639 (PMC11432034; doi:10.3390/insects15090639)
Supplement: Supplementary file 1 [file insects-15-00639-s001.zip › supplemental data.pdf]

Table S1: sample information.

#	species	collection location	other ID	ank2 allele closest	pK1 allele closest	wPip group	Wol abdomen	Wol thorax	Collection date	Latitude	longitude
1	Culex quiquefasciatus	Mali	2021-10	A	B	wPip-III	6,99E+05	3,61E+04			
2	Culex quiquefasciatus	Mali	2021-14	A	B	wPip-III	7,52E+05	9,70E+04			
3	Culex quiquefasciatus	Mali	2021-15				1,62E+02	2,15E+01			
4	Culex quiquefasciatus	Mali	2021-17	A	B	wPip-III	3,73E+05	4,31E+04			
5	Culex quiquefasciatus	Mali	2021-19	A	B	wPip-III	1,75E+06	9,89E+03			
6	Culex pipiens f molestus	Nickelmyntsgatan 11	2019_3	B	A	wPip-I	2,29E+05	1,35E+02	2019-05-02	57,67327	11,93056
7	Culex pipiens f molestus	Televisionsgatan 9, Västra Frölunda	2019_13	B	A	wPip-I	6,64E+05	1,06E+02	2019-09-12	57,659568	11,924779
8	Culex pipiens f molestus	Drammen, Norge	2020-1	C	D	wPip-IV	6,85E+05	8,97E+02	2020-01-24		
9	Culex pipiens f molestus	Drammen, Norge	2020-6	C			1,18E+05	2,19E+02	2020-01-24		
10	Culex pipiens f pipiens	Filipshyttan, Närke	2020-48	B	C*	wPip-II	1,15E+06	4,01E+04	2020-12-31		
11	Culex pipiens f pipiens	Filipshyttan, Närke	2020-49		C*	wPip-II	8,81E+05	4,36E+04	2020-12-31		
12	Culex pipiens f pipiens	Tellgrensgatan 2B, Gbg	4;2018	B	C*	wPip-II	8,13E+05	1,00E+05		57.695436	11.924395
13	Culex pipiens f molestus	Kaplandsgatan 38, Gbg	17;2018	B	A	wPip-I	3,19E+05	1,44E+02		57.6709834	11.9330281
14	Culex pipiens molestus hybrid	Kaplandsgatan 38, Gbg	18;2018	B	A	wPip-I	3,18E+05	3,07E+01		57.6709834	11.9330281
15	Culex pipiens f molestus	Köinge 2050; Hörby	55;2018	B	A	wPip-I	1,45E+04	4,03E+02		55,833974	13,719499
16	Culex pipiens f molestus	Köinge 2050; Hörby	56;2018	B	A	wPip-I	2,58E+04	6,86E+02		55,833974	13,719499
17	Culex pipiens f molestus	Mörlandavägen 5; sollebrunn	66:2018	B	A	wPip-I	2,63E+04	0,00E+00		58,11242	12,562265
18	Culex pipiens f molestus	Mörlandavägen 5; sollebrunn	67;2018	B	A	wPip-I	1,09E+05	8,88E+01		58,11242	12,562265
19	*Culex torrentium*	Simrishamn	27;2018				0,00E+00	0,00E+00		55.556637	14.352959
20	*Culex torrentium*	Simrishamn	31;2018				0,00E+00	0,00E+00		55.556637	14.352959
21	*Culex torrentium*	Simrishamn	35;2018				0,00E+00	0,00E+00		55.556637	14.352959
22	*Culex torrentium*	Simrishamn	36;2018				0,00E+00	0,00E+00		55.556637	14.352959
23	*Culex pipiens*	Simrishamn	28;2018	B	C*	wPip-II	4,31E+05	7,27E+04		55.556637	14.352959
24	*Culex pipiens*	Simrishamn	29;2018	B	C*	wPip-II	1,63E+06	3,44E+04		55.556637	14.352959
25	*Culex pipiens*	Simrishamn	30;2018	B	C*	wPip-II	9,10E+04	4,98E+01		55.556637	14.352959
26	*Culex modestus*	Vellinge	131h				0,00E+00	0,00E+00	2017-08-20	55.384434	12.816393
27	*Culex modestus*	Vellinge	131h				1,22E+01	4,87E+00	2017-08-20	55.384434	12.816393
28	*Culex modestus*	Vellinge	133a				0,00E+00	0,00E+00	2017-07-28-31	55.384434	12.816393
29	*Culex modestus*	Vellinge	133b				10,63736612	0	2017-07-28-31	55.384434	12.816393
30	*Culex modestus*	Vellinge	136e				19,75765519	64,20074376	2017-07-27	55.384434	12.816393
31	*Culex modestus*	Vellinge	137j				0	39,68832452	2017-07-28	55.384434	12.816393
32	Culex territans	Mora	139b				0	0		61.033804	14.525026
33	Culex pipiens f pipiens	Mora	139c	B	C*	wPip-II	573867,0938	16,97194684		61.033804	14.525026
34	Culex pipiens f molestus	Burlöv	12a	B	A	wPip-I	212805,0206	13,98599926	2017-06-19	55.636584	13.062753
35	Culex territans	Helsingborg	112				0	0		56.04401	12.742729
36	Culex pipiens f pipiens	Filipshyttan, Närke	2020-45				6208,388106	1176,0908	2020-12-31	59.331799	14.989772
37	Culex pipiens f pipiens	Filipshyttan, Närke	2020-46	B	C*	wPip-II	197641,7608	0	2020-12-31	59.331799	14.989772
38	Culex pipiens f pipiens	Filipshyttan, Närke	2020-47	B			314660,0517	847,1743855	2020-12-31	59.331799	14.989772
39	Culex torrentium	Trelleborg	21				0	0		55.36237	13.212191
40	Culex torrentium	Karlskrona	39				0	0	2017-07-14	56.304752	15.655006
41	Culex torrentium	Malmö	57				0	0		55.590642	13.018583
42	Culex torrentium	Uppsala	68				0	0		59.876496	17.623683
43	Culex torrentium	Mölndal	73				0,00E+00	0,00E+00	2017-07-26	57.667114	12.046808
44	Aedes vexans	Eslöv	43				0,00E+00	0,00E+00	2017-08-26	55.851875	13.299292
45	Aedes vexans	Eslöv	43				0,00E+00	9,77E+01	2017-08-26	55.851875	13.299292
46	Aedes vexans	Eslöv	43				0,00E+00	2,39E+01	2017-08-26	55.851875	13.299292
47	Aedes vexans	Eslöv	43				0,00E+00	0,00E+00	2017-08-26	55.851875	13.299292
48	Aedes vexans	Eslöv	43				0,00E+00	0,00E+00	2017-08-26	55.851875	13.299292
49	Aedes vexans	Vellinge	153				0,00E+00	0,00E+00	2017-08-22	55.384434	12.816393
50	Aedes vexans	Simrishamn	116d				0,00E+00	0,00E+00	2017-08-31	55.556637	14.352959
51	Aedes vexans	Simrishamn	116d				0,00E+00	1,55E+01	2017-08-31	55.556637	14.352959
52	Aedes vexans	Simrishamn	117c				0,00E+00	0,00E+00	2017-09-07	55.556637	14.352959
53	Aedes vexans	Simrishamn	118b				0,00E+00	0,00E+00	2017-08-30	55.556637	14.352959
54	Aedes vexans	Simrishamn	118b				0,00E+00	0,00E+00	2017-08-30	55.556637	14.352959
55	Aedes vexans	Vellinge	133aa				0,00E+00	1,34E+01	2017-07-28	55.384434	12.816393
56	Aedes vexans	Vellinge	133aa				0,00E+00	0,00E+00	2017-07-28	55.384434	12.816393
2020_12	Culex pipiens f molestus	Drammen, Norge		C	D	wPip-IV					
2020_16	Culex pipiens f molestus	Drammen, Norge		C	D	wPip-IV					
2020_17	Culex pipiens f molestus	Drammen, Norge		C							
2020_18	Culex pipiens f pipiens	Drammen, Norge		other	C*	wPip-II					
2020_5	Culex pipiens f pipiens	Drammen, Norge	2020-5	B							
2020_7	Culex pipiens hybrid	Drammen, Norge		other	D	wPip-IV					
2020_8	Culex pipiens f pipiens	Drammen, Norge		B	C	wPip-II					
2021-91	Culex pipiens f molestus	Blåsutgatan 13, Gbg					393997,2118	22,45143214			

LHT-01	Culex pipiens hybrid	Brookwood		A	C	wPip-II	43391,90413	48908,40773	
LHT-02	Culex pipiens hybrid	Brookwood		A	C	wPip-II	140136,9425	351639,7788	
LHT-03	Culex pipiens hybrid	Brookwood		A	C	wPip-II	89129,40075	95325,64511	
LHT-04	Culex pipiens hybrid	Brookwood		A	C	wPip-II	282639,1822	63965,48507	
LHT-05	Culex pipiens hybrid	Brookwood		A	C	wPip-II	456837,277	171816,1237	
LHT-06	Culex pipiens hybrid	Brookwood		A	C	wPip-II	376473,9756	80046,89557	
LHT-07	Culex pipiens f molestus	Brookwood		A	C	wPip-II	43588,02173	43588,02173	
LHT-08	Culex pipiens f molestus	Brookwood		A	C	wPip-II	38451,99817	38451,99817	
LHT-09	Culex pipiens f molestus	Brookwood		A	C	wPip-II	29870,05687	78481,57404	
LHT-10	Culex pipiens f molestus	Brookwood		A	C	wPip-II	94405,3437	94405,3437	
LHT-11	Culex pipiens f molestus	Brookwood		A	C	wPip-II	43237,91532	62155,51587	
LHT-12	Culex pipiens f molestus	Brookwood		A	C	wPip-II	909193,3523	92792,34745	
LHT-13	Culex pipiens f pipiens	Brookwood		A	C	wPip-II	118793,3576	139131,1211	
LHT-14	Culex pipiens f pipiens	Brookwood		A	C	wPip-II	56481,81782	69876,0747	
LHT-15	Culex pipiens f pipiens	Brookwood		A	C	wPip-II	42792,65399	62370,38657	
LHT-16	Culex pipiens f pipiens	Brookwood		A			84966,8696	34318,28241	
LHT-17	Culex pipiens f pipiens	Brookwood		A	C	wPip-II	85929,20014	76758,54678	
LHT-18	Culex pipiens f pipiens	Caldbeck					108133,5669	112015,6581	
LHT-19	Culex pipiens f pipiens	Caldbeck		A	C	wPip-II	24300,75575	179450,1906	
LHT-20	Culex pipiens f pipiens	Caldbeck		A	C	wPip-II	66337,62711	94106,64987	
LHT-21	Culex pipiens f pipiens	Caldbeck		A	C	wPip-II	18326,34462	67696,21829	
LHT-22	Culex pipiens f pipiens	Caldbeck			C	wPip-II	192473,3676	163562,762	
775	Culex pipiens f pipiens	Fermo / FERMO - (FM)					8,622266755		
776	Culex pipiens f pipiens	Fermo / FERMO - (FM)					17603,9337		
777	Culex pipiens f pipiens	Fermo / FERMO - (FM)					23963,82172		
779	Culex pipiens f pipiens	Fermo / FERMO - (FM)					20862,35802		
781	Culex pipiens f pipiens	Fermo / FERMO - (FM)					51353,05311		
901	Culex pipiens f pipiens	Fermo / FERMO - (FM)					5816622,835		
902	Culex pipiens f pipiens	Fermo / FERMO - (FM)					1274,474445		
903	Culex pipiens f pipiens	Fermo / FERMO - (FM)					11218,94645		
904	Culex pipiens f pipiens	Fermo / FERMO - (FM)					202408,7343		
905	Culex pipiens f pipiens	Fermo / FERMO - (FM)					124920,8213		
906	Culex pipiens f pipiens	Fermo / FERMO - (FM)		A	B	wPip-III	2510373,594		
908	Culex pipiens f pipiens	Fermo / FERMO - (FM)		A	E	wPip-V	154524,3328		
909	Culex pipiens f pipiens	Fermo / FERMO - (FM)		A			270078,0968		
GBG-04	Culex pipiens f pipiens	Gothenburg	A20F	B					2021-07-12
GBG-07	Culex pipiens f pipiens	Gothenburg	A20F	B					2021-07-12
GBG-103	Culex pipiens f pipiens	Gothenburg	A2H	B					2021-08-12
GBG-108	Culex pipiens f pipiens	Gothenburg	A1F	ED-special	C*	wPip-II			2021-08-24
GBG-110	Culex pipiens f pipiens	Gothenburg	A1F	ED-special					2021-08-24
GBG-111	Culex pipiens f pipiens	Gothenburg	A1F	B					2021-08-24
GBG-112	Culex pipiens f pipiens	Gothenburg	A1F	B					2021-08-24
GBG-114	Culex pipiens f pipiens	Gothenburg	A1F	B	C*	wPip-II			2021-08-24
GBG-119	Culex pipiens f pipiens	Gothenburg	A2F	B					2021-08-26
GBG-16	Culex pipiens f pipiens	Gothenburg	A2I	B					2021-07-22
GBG-19	Culex pipiens f pipiens	Gothenburg	A2I	B					2021-07-22
GBG-21	Culex pipiens f pipiens	Gothenburg	A2I	B					2021-07-22
GBG-27	Culex pipiens f pipiens	Gothenburg	A17F	B					2021-07-22
GBG-28	Culex pipiens f pipiens	Gothenburg	A17F	B					2021-07-22
GBG-29	Culex pipiens f pipiens	Gothenburg	A17F	B					2021-07-22
GBG-31	Culex pipiens f pipiens	Gothenburg	A1F	B					2021-07-22
GBG-35	Culex pipiens f pipiens	Gothenburg	A1F	B					2021-07-22
GBG-36	Culex pipiens f pipiens	Gothenburg	A1F	B					2021-07-22
GBG-37	Culex pipiens f pipiens	Gothenburg	A1F	B					2021-07-22
GBG-47	Culex pipiens f molestus	Gothenburg	A1F	B					2021-07-22
GBG-62	Culex pipiens f molestus	Gothenburg	A17F	B	A	wPip-I			2021-08-25
GBG-65	Culex pipiens f molestus	Gothenburg	A3F	B					2021-07-09
GBG-66	Culex pipiens f molestus	Gothenburg	A8F	B	A	wPip-I			2021-07-09
GBG-68	Culex pipiens f pipiens	Gothenburg	A13F	B					2021-07-13
GBG-76	Culex pipiens f pipiens	Gothenburg	A15F	B					2021-07-13
GBG-79	Culex pipiens f pipiens	Gothenburg	A2H	B	C*	wPip-II			2021-07-13
GBG-81	Culex pipiens f pipiens	Gothenburg	A2H	B					2021-07-13

GBG-84	Culex pipiens f molestus	Gothenburg	A2F	B	A	wPip-I			2021-07-13
GBG-90	Culex pipiens f molestus	Gothenburg	A2K	B					2021-06-30
GBG-97	Culex pipiens f pipiens	Gothenburg	A15F	B					2021-07-26
2021-67	Culex pipiens f pipiens	Kennedygatan 6B Gothenburg					500,7470648	32,80480741	
616	Culex pipiens f molestus	Rome / Montelibretti (RM)					244487,9242	22,99423034	
617	Culex pipiens f molestus	Rome / Montelibretti (RM)					4782,421883	29,22330603	
618	Culex pipiens f molestus	Rome / Montelibretti (RM)		A	B	wPip-III	199346,9074	23,32459686	
619	Culex pipiens f molestus	Rome / Montelibretti (RM)					330,469108	33,50811215	
620	Culex pipiens f molestus	Rome / Montelibretti (RM)		A	B	wPip-III	38882,51275	25,94570782	
621	Culex pipiens f molestus	Rome / Montelibretti (RM)		A	B	wPip-III	14220,08323	27,49544724	
622	Culex pipiens f molestus	Rome / Montelibretti (RM)		A	B	wPip-III	36652,59634	25,99844857	
623	Culex pipiens f molestus	Rome / Montelibretti (RM)		A	B	wPip-III	5088,626476	30,19111473	
624	Culex pipiens f molestus	Rome / Montelibretti (RM)		A	B	wPip-V	51208,17199	25,46540849	
625	Culex pipiens f molestus	Rome / Montelibretti (RM)					9950,506003	28,05619035	
626	Culex pipiens f molestus	Rome / Montelibretti (RM)		A	B	wPip-III	115161,5179	24,37230147	
627	Culex pipiens f molestus	Rome / Montelibretti (RM)					33,81285821	37,37267558	
628	Culex pipiens f molestus	Rome / Montelibretti (RM)					234167,0298	23,10819222	
629	Culex pipiens f molestus	Rome / Montelibretti (RM)					5262,430408	29,06768167	
630	Culex pipiens f molestus	Rome / Montelibretti (RM)					2303,551995	30,70192184	
631	Culex pipiens f molestus	Rome / Montelibretti (RM)					12,94416303	36,74265981	
632	Culex pipiens f molestus	Rome / Montelibretti (RM)					877866,4553	23,70588287	
633	Culex pipiens f molestus	Rome / Montelibretti (RM)					3493,817399	30,15359404	
634	Culex pipiens f molestus	Rome / Montelibretti (RM)					86503,25135	26,47799773	
635	Culex pipiens f molestus	Rome / Montelibretti (RM)		A	B	wPip-III	1712,992791	30,99742259	
121	Culex pipiens f molestus	Rome / Roma - Aurelio		A	B	wPip-III	4131980,391	22,00312046	
125	Culex pipiens f molestus	Rome / Roma - Aurelio		A	B	wPip-III	1352610,161	23,32571647	
127	Culex pipiens f molestus	Rome / Roma - Aurelio		A	B	wPip-III	487676,4566	24,39638051	
128	Culex pipiens f molestus	Rome / Roma - Aurelio		A	B	wPip-III	240786,0815	25,37549661	
129	Culex pipiens f molestus	Rome / Roma - Aurelio		A	B	wPip-III	257433,7242	25,19458375	
130	Culex pipiens f molestus	Rome / Roma - Aurelio					3876512,213	22,02275919	
131	Culex pipiens f molestus	Rome / Roma - Aurelio					18452,84257	28,2101982	
132	Culex pipiens f molestus	Rome / Roma - Aurelio					27,69137515	35,88405246	
133	Culex pipiens f molestus	Rome / Roma - Aurelio					308,9752541	33,01176619	
134	Culex pipiens f molestus	Rome / Roma - Aurelio					14704,61219	28,537697	
135	Culex pipiens f molestus	Rome / Roma - Aurelio			B	wPip-III	557,523665	32,34335026	
136	Culex pipiens f molestus	Rome / Roma - Aurelio					4984,024397	29,93434199	
137	Culex pipiens f molestus	Rome / Roma - Aurelio					6314,929736	29,49028903	
138	Culex pipiens f molestus	Rome / Roma - Aurelio					8598,237124	29,23152409	
139	Culex pipiens f molestus	Rome / Roma - Aurelio					6818,504754	29,38246132	
140	Culex pipiens f molestus	Rome / Roma - Aurelio					4842,84546	30,00750554	
984	Culex pipiens f pipiens	Rome / Roma - Insugherata					1074,264231	31,54539447	
985	Culex pipiens f pipiens	Rome / Roma - Insugherata					3189,129738	29,94863401	
990	Culex pipiens f pipiens	Rome / Roma - Insugherata					24,01629336	37,31580586	
991	Culex pipiens f pipiens	Rome / Roma - Insugherata					1323,498851	31,22361796	
992	Culex pipiens f pipiens	Rome / Roma - Insugherata					23,28839615	37,46239606	
993	Culex pipiens f pipiens	Rome / Roma - Insugherata					7109,04981	28,73565758	
997	Culex pipiens f pipiens	Rome / Roma - Insugherata					27,953028	37,00687319	
998	Culex pipiens f pipiens	Rome / Roma - Insugherata					51,21243649	36,11685858	
999	Culex pipiens f pipiens	Rome / Roma - Insugherata					403,2372954	33,05829486	
1000	Culex pipiens f pipiens	Rome / Roma - Insugherata					442,1862233	32,87271019	
1002	Culex pipiens f pipiens	Rome / Roma - Insugherata					186,4670906	34,22557469	
2021-90	Culex pipiens f molestus	Rubelgatan 6, Gothenburg					153544,2974		
2022-07	Culex pipiens f molestus	Schiphol Airport (Strain)					85929,20014	76758,54678	
2022-08	Culex pipiens f molestus	Schiphol Airport (Strain)					2956,251069	1911,774407	
2022-06	Culex pipiens f molestus	Schiphol Airport (Strain)					3393,978659	1650,079396	
2021-93	Culex pipiens f molestus	Strandridaregatan, Gbg		B	A	wPip-I	301279,0952		
2022-09	Culex quinquefasciatus	Thai Strain(London)					67064,86715	14135,72981	
2022-10	Culex quinquefasciatus	Thai Strain(London)					88877,15951	9029,812301	
2022-11	Culex quinquefasciatus	Thai Strain(London)					57787,76031	8580,103406	
2021-81	Culex pipiens f molestus	Uddevalla		B	A	wPip-I	80495,76394		
2021-82	Culex pipiens f molestus	Uddevalla					73,92396251		
2021-83	Culex pipiens f molestus	Uddevalla		B	A	wPip-I	119765,1745		

2021-84	Culex pipiens f molestus	Uddevalla	99,53153217	
2022-03	Culex pipiens f pipiens	Wageningen(strain)	85391,893	5086,603825
2022-04	Culex pipiens f pipiens	Wageningen(strain)	231464,0468	172933,6714
2022-05	Culex pipiens f pipiens	Wageningen(strain)	469345,9497	1404,279085
