# Supplementary material for: European Culex pipiens Populations Carry Different Strains of Wolbachia pipientis
Source: Insects. 2024 Aug 26;15(9):639. doi: 10.3390/insects15090639 (PMC11432034; doi:10.3390/insects15090639)
Supplement: Supplementary file 1 [file insects-15-00639-s001.zip › supplemental-Figure S1.pdf]

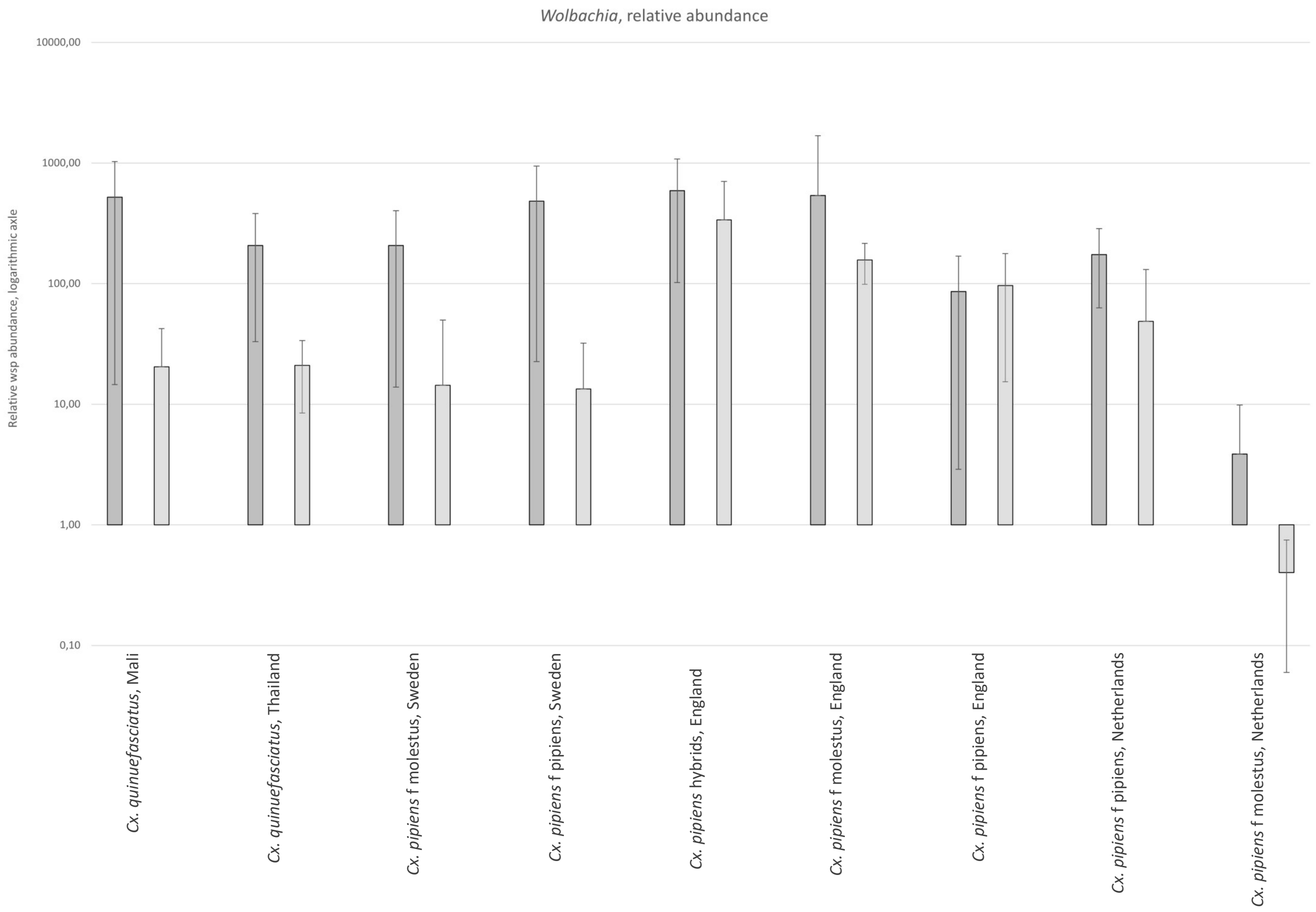


Supplemantal Figure S1. *Wolbachia* abundance in specimens. Abundance measured with qPCR against *Wolbachia* wsp gene. For each population, dark grey bars are mean of abdomen samples and light grey bars are mean of rest of body samples. Error bars are standard variation of the mean. Y axis is logarithmic abundance relative to a standard sample. The levels were tested statistically using Students t-test and were found to be significantly higher in the abdomen than in the rest of the body in *Cx. quinquefasciatus* from Mali, in Swedish *Cx. pipiens* f pipiens and Swedish *Cx. pipiens* f molestus while there was no significant difference in the other populations.
